# Frequency and severity of irritable bowel syndrome in cigarette smokers, Turkey 2019

**DOI:** 10.18332/tid/145925

**Published:** 2022-03-07

**Authors:** Melike Mercan Başpınar, Okcan Basat

**Affiliations:** 1 Department of Family Medicine, University of Health Sciences Gaziosmanpaşa Training and Research Hospital, Istanbul, Turkey

**Keywords:** irritable bowel syndrome, nicotine dependence, Rome IV criteria, smoking

## Abstract

**INTRODUCTION:**

Cigarette smoking has recently been associated with several gastrointestinal symptoms, and smoking cessation has been recommended as a lifestyle change strategy for irritable bowel syndrome (IBS). This study assessed the prevalence of IBS in cigarette smokers based on the Rome IV criteria, the severity of nicotine dependence, and the effect of smoking cessation in smokers with IBS.

**METHODS:**

This prospective study included 371 smokers who attended smoking cessation treatment at family medicine clinics in a tertiary hospital between January and April 2019, in Turkey. Data on demographic characteristics, IBS status according to the Rome IV criteria, and Fagerström test for nicotine dependence (FTND) scores were collected during face-to-face interviews.

**RESULTS:**

The mean patient age was 40.7 ± 11.96 years. Out of the total patients, 29.4% were heavy smokers, and 18.1% had IBS. There was a significant difference in age (p=0.03), duration of smoking (p=0.05), FTND score (p=0.02), and sex (p<0.001) between those with and without IBS. Logistic regression analyses identified female sex as a predictor of IBS in smokers (adjusted odds ratio, AOR=1.78; 95% CI: 1.18–2.69; p=0.006). At follow-up at 1 year, IBS(+) smokers who had quit smoking showed decreased gastrointestinal symptoms (p=0.035).

**CONCLUSIONS:**

FTND score was higher in IBS(+) smokers than in IBS(-) smokers. Smoking cessation ameliorated gastrointestinal symptoms but did not affect IBS status.

## INTRODUCTION

Irritable bowel syndrome (IBS) is a chronic functional disorder of the gastrointestinal (GI) tract that affects about 11% worldwide^1^. In Turkey, the reported prevalence of IBS varied from 6.3–19.1% before the publication of the Rome IV criteria^2-5^. Introduction of the Rome IV criteria in May 2016^6,7^ led to a decrease in IBS detection rates^8^, likely because the revised criteria were better able to differentiate true IBS^7^.

Smoking cessation has been recommended as a lifestyle change strategy for IBS management^9-11^. Current or former smoking has been associated with several GI symptoms, including abdominal pain, constipation, and bloating^12^. Moreover, studies have shown that smoking contributes to GI disorders by decreasing blood flow, having adverse effects on the mucosa, and releasing free radicals that slow cell proliferation^12,13^.

Multiple reports have linked IBS pathogenesis with dysbiosis. This condition refers to the decrease/loss of microbial diversity and richness, owing to the changes from commensal bacteria to pathogens in the human gut^14^. A nicotine/smoking dose-response association has been observed for some bacterial taxa in gut flora, with an increasing mean relative abundance of specific taxa as cigarette packs per day increased^15^.

Thus, we hypothesized that nicotine dependence would be more severe in IBS(+) smokers than in IBS(-) smokers. This study aimed to assess the presence of nicotine dependence and IBS in treatment-seeking cigarette smokers using the Rome IV criteria and the effect of smoking cessation on IBS and GI symptoms.

## METHODS

### Subjects

This study had a prospective observational design and included 371 current smokers (aged ≥18 years) who sought smoking cessation treatment at family medicine clinics in a training and research hospital between January and April 2019.

Patients were excluded from participation in the study if any of the following were present: red flag symptoms in recent months, such as weight loss, anemia, or hematochezia; a previous diagnosis of another GI disorder (e.g. functional diarrhea, functional constipation, ulcerative colitis, or Crohn’s disease); use long-term medication known to have adverse GI-related side effects (e.g. opioid analgesics, nonsteroidal anti-inflammatory drugs, anti-allergic agents, calcium channel blockers, beta-blockers, tricyclic antidepressants and selective serotonin reuptake inhibitors or anxiolytics); or systemic disease associated with bowel habit (e.g. a psychiatric diagnosis, thyroid dysfunction, food allergy, diabetes mellitus, chronic renal failure, multiple sclerosis, or systemic lupus erythematosus).

Data on demographic characteristics, IBS status according to the Rome IV criteria, and Fagerström test for nicotine dependence (FTND) scores were collected during face-to-face interviews. Demographic data included patient sex and age, marital status, education level, and personal and family medical history.

### Diagnosis of IBS by the Rome IV criteria

IBS was diagnosed if the patient reported recurrent abdominal pain occurring on average at least one day per week in the last three months that was associated with two or more of the following criteria: abdominal pain related to defecation; change in stool frequency; change in the form (appearance) of stool; and symptom onset at least six months before diagnosis^16^.

### Assessment of nicotine dependence level

The nicotine dependence level (NDL) was assessed using the FTND. The reliability of the Turkish version of this tool was confirmed in 2004^17^. Nicotine dependence was classified as mild (0–4 points), moderate (5–7 points), or heavy (8–10 points).

### Follow-up

Study participants diagnosed with IBS were followed up one year later by telephone. Successful smoking cessation was defined as not smoking during the last six months. At the telephone call follow-up after one year, 67 smokers with IBS who had received smoking cessation treatment were questioned about their GI symptoms, presence of IBS, and smoking status.

### Sample size calculation

The required sample size was estimated to be 363 (min 272) based on the prevalence of IBS in the general population being estimated to be 13% with an estimated error of ± 4%, Type 1 error (alpha) 5% (two-tailed), power 95%, and allowing for a 25% dropout rate.

### Statistical analysis

The database was built in Excel. The data are shown as mean and standard deviation or as number and percentage, as appropriate. Categorical data and continuous data were compared using the chi-squared test and Mann-Whitney U test, respectively. The statistical analysis was performed using NCSS 10 software (2015; Kaysville, UT, USA). A p≤0.05 was considered statistically significant.

## RESULTS

**Figure 1:**
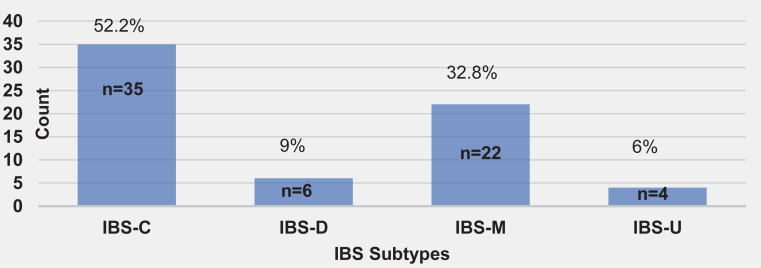
Distribution of IBS subtypes. IBS, irritable bowel syndrome; IBS-C, constipation-predominant irritable bowel syndrome; IBS-D, diarrhea-predominant irritable bowel syndrome; IBS-M, mixed irritable bowel syndrome; IBS-U, unspecified irritable bowel syndrome

The sample comprised 371 patients (41.2% female, 58.8% male) of mean age 40.7 ± 11.96 years. Current smokers who had experienced abdominal pain for at least one day per week in the last three months (34.5%) were selected for investigation using the Rome IV criteria. At the next monthly visit, 26.6% were confirmed to have recurrent weekly abdominal pain. IBS was diagnosed in 18.1% of all cases using the Rome IV criteria. IBS-C was the most common subtype (52.2%), followed by IBS-M (32.8%), IBS-D (9%), and IBS-U (6%). Figure 1 shows the distribution of the IBS subtypes.

When asked: ‘How do you think smoking affects your defecation?’, 38% of respondents said that smoking helped, and 52.8% said that smoking had no effect.

Constipation, diarrhea, and abdominal bloating, were reported by 45.6%, 23.7%, and 52% of respondents, respectively. Eighty-five per cent of smokers with constipation, 42% with diarrhea, and 94% with abdominal bloating were diagnosed with IBS.

The NDL was heavy in 109 (29.4%), moderate in 173 (46.6%), and mild in 89 patients (24.0%); 197 (53.1%) patients with IBS smoked more than 20 cigarettes/day. The mean duration of smoking was 22.00 ± 12.00 years. The mean number of pack-years of smoking was 28.47 ± 19.92, and the mean FTND score was 5.94 ± 2.31.

### Comparison of continuous data in the IBS(+) and IBS(-) smokers

There was a statistically significant age difference (p=0.03), duration of smoking (p=0.05), and FTND score (p=0.02) between patients with and without IBS. Compared with IBS(-) smokers, the IBS(+) smokers had a higher mean nicotine dependence score, were older, and had been smokers for longer (Table 1).

**Table 1:** Comparison of continuous data between smokers with IBS(+) and without IBS(-), Turkey 2019 (N=371)

*Variable*	*Total mean ± SD*	*IBS(+) mean ± SD*	*IBS(-) mean ± SD*	*p*
**Age** (years)	40.70 ± 11.96	43.20 ± 10.96	40.10 ± 12.12	0.03*
**Cigarettes** (per day)	24.33 ± 11.29	24.37 ± 12.62	24.32 ± 11.00	0.67
**Cigarette** (pack-years)	28.47 ± 19.92	30.99 ± 19.97	27.90 ± 19.90	0.17
**Duration of smoking** (years)	22.00 ± 12.00	25.61 ± 10.84	23.12 ± 12.24	0.05*
**Age of starting smoking** (years)	16.90 ± 4.76	17.45 ± 4.92	16.76 ± 4.72	0.36
**FTND score**	5.94 ± 2.31	6.49 ± 2.40	5.82 ± 2.28	0.02*

* p<0.05; Mann-Whitney U test.

FTND: Fagerström test for nicotine dependence. IBS: irritable bowel syndrome.

### Comparison of categorical data in the IBS(+) and IBS IBS(-) smokers

The prevalence of IBS was higher in women than in men (27.5% vs 11.5%; p<0.001). No significant difference was observed between the IBS(+) and IBS(-) smokers in marital status, education level, income level, or family history of GI symptoms or functional GI disorders (FGIDs) (Table 2). Furthermore, there was no relationship between the presence of IBS and the severity of nicotine dependence (p=0.14). The presence of IBS in the group with heavy NDL was similar to that in the other NDL groups.

**Table 2:** Comparisons of categorical data between smokers with IBS(+) and without IBS(-), Turkey 2019 (N=371)

*Variable*	*IBS(+) n (%)*	*IBS(-) n (%)*	*p*
**Sex**			<0.001*
Male	25 (37.3)	193 (63.5)	
Female	42 (62.7)	111 (36.5)	
**Marital status**			0.24
Married	57 (85.1)	236 (77.6)	
Single	10 (14.9)	68 (22.4)	
**Education level**			0.10
Basic education	44 (65.7)	157 (51.6)	
High school	14 (20.9)	81 (26.6)	
University	9 (13.4)	66 (21.7)	
**Income level**			0.07
Low	33 (49.3)	104 (34.2)	
Medium	20 (29.9)	114 (37.5)	
High	14 (20.9)	86 (28.3)	
**Cigarettes** (per day)			0.03*
≤10	26 (6.0)	22 (7.2)	
11–20	148 (46.3)	117 (37.5)	
21–30	125 (19.4)	112 (36.8)	
≥31	72 (28.4)	53 (17.4)	
**Nicotine dependence level**			0.14
Mild	12 (17.9)	77 (25.3)	
Moderate	29 (43.3)	144 (47.4)	
Heavy	26 (38.8)	83 (27.3)	
**Family history** (GI symptoms or FGIDs)			0.34
(+)	20 (29.9)	71 (23.4)	
(-)	47 (70.1)	233 (76.6)	

* p<0.05, chi-squared test.

FGIDs: functional gastrointestinal disorders. GI: gastrointestinal. IBS: irritable bowel syndrome.

### Logistic regression analysis

**Figure 2:**
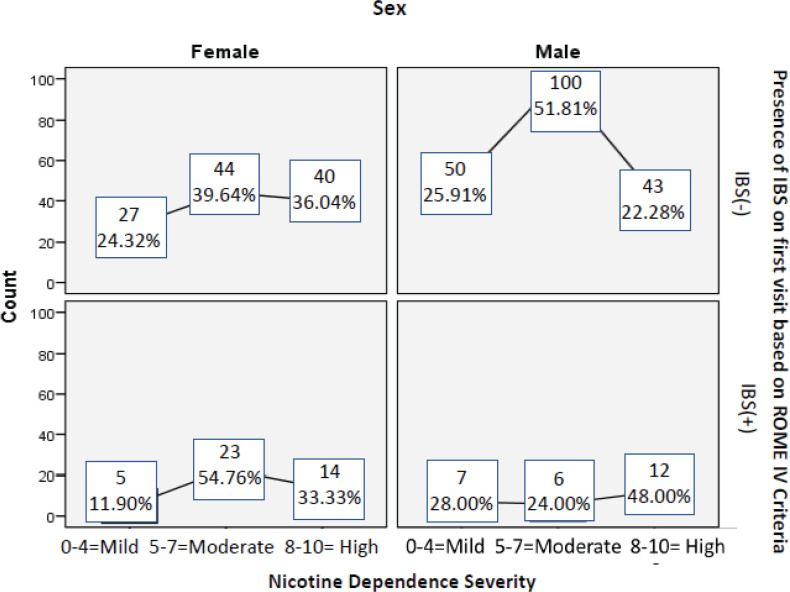
Nicotine dependence level and IBS according to sex. IBS, irritable bowel syndrome

Table 3 shows the predictors for IBS in smokers. Logistic regression analysis showed that the odds of having IBS were 1.78 times higher in women than in men. Female sex was the only independent risk factor for IBS(+) in smokers (AOR=1.78; 95% CI: 1.18–2.69; p=0.006). Most women with IBS had a moderate NDL, while most men with IBS had a high NDL (Figure 2).

**Table 3:** Predictors of IBS identified by logistic regression analysis, Turkey 2019 (N=371)

*Variable*	*p*	*OR*	*95 % CI*	
			*Lower*	*Upper*
**Age** (years)	0.80	1.01	0.96	1.06
**Sex**				
Male (Ref.)		1	-	-
Female	0.006*	1.78	1.18	2.69
**Education level**				
Basic education (Ref.)		1	-	-
High school	0.29	1.28	0.81	2.03
University	0.97	1.01	0.63	1.63
**Income**				
Low income (Ref.)		1	-	-
Medium income	0.36	0.77	0.44	1.34
High income	0.98	0.99	0.63	1.57
**Pack-years of smoking**	0.45	0.99	0.97	1.01
**FTND score**	0.20	1.25	0.89	1.76
**Years of smoking**	0.62	1.02	0.96	1.07
**NDL**				
Mild (Ref.)		1	-	-
Moderate	0.55	1.39	0.47	4.11
Heavy	0.58	0.89	0.61	1.32

* p<0.05.

Logistic regression analysis. FTND: Fagerström test for nicotine dependence. NDL: nicotine dependence level. OR: odds ratio.

### Evaluation of smoking cessation and IBS at follow-up at 1 year

**Figure 3:**
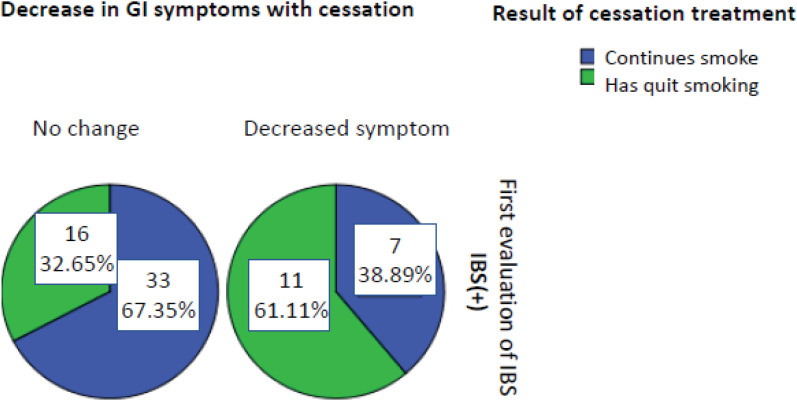
Effect of successful smoking cessation on GI symptoms in IBS(+) smokers. GI, gastrointestinal; IBS, irritable bowel syndrome

Cessation treatment was successful in 27 (40.3%) of 67 smokers with IBS who had received smoking cessation treatment, of whom 62 had no change in their recurrent abdominal pain and were again evaluated as being IBS(+). In contrast, five no longer had recurrent abdominal pain and were diagnosed as IBS(-). The effect of smoking cessation on IBS prognosis in the first year was not statistically significant (p=0.063, McNemar’s test). However, 18 (26.9%) of the 67 IBS(+) smokers reported a decrease in GI symptoms, such as abdominal bloating, constipation, and diarrhea, in the first year after smoking cessation (Figure 3). Furthermore, there was a significant difference in GI symptoms between former and current smokers after smoking cessation (p=0.035).

## DISCUSSION

Primary care physicians have an important role in the management of IBS. It was found that 90% of patients with medically diagnosed IBS had sought treatment from a primary care physician, whereas only 28% were managed by a gastroenterologist. In this study, we found no relationship between IBS and NDL in cigarette smokers but found that the nicotine dependence score was higher in IBS(+) than in IBS(-) smokers. We also found that although there was a 27% decrease in GI symptoms at follow-up at 1 year, there was no significant effect of smoking cessation on IBS diagnosis.

Unlike in the Rome III criteria, in which patients with abdominal discomfort could be diagnosed with IBS, Rome IV lists abdominal pain as the main criterion. Its criteria have recently been updated to include unspecified functional bowel disorder. The effect of restricting IBS diagnosis to abdominal pain with IBS-C in the Rome IV criteria is unknown^18^. A population-based study suggests that abdominal bloating substantially impacts patients’ daily lives with IBS-C^19^. In this study, abdominal bloating was 52% overall, and 94% of smokers with abdominal bloating were diagnosed with IBS.

Former smoking is often associated with functional bloating and functional constipation^12^. In this study, all patients were current smokers on the first visit. Hence, the high prevalence of constipation and IBS-C (52.2%) was unexpected, suggesting that symptoms of constipation may be more common in current smokers than in the general population. Still, Talley et al.^20^ found that heavy smokers had significantly higher odds of diarrhea, flatus, urgency than non-smokers. Additionally, smoking was associated with a significantly increased risk of IBS-diarrhea, while this only applied to the highest exposure category (20 or more cigarettes per day), there was a trend for a dose-response relationship. On the other hand, there was no relationship between IBS-C and IBS-M^20^. A systematic review found that the reported prevalence of constipation was 2.6–26.9%^21^. Another study found a significant association between current and former smoking and functional GI symptoms, particularly functional abdominal pain, bloating, and constipation^12^. The former smokers, in our study, reported a significant decrease in GI symptoms, which indicates that smoking cessation improves GI symptoms in IBS(+) smokers.

IBS is more common in women, and female sex hormones have contributed to this predominance^22-25^. In one study, digestive symptoms were noticed at least every two months in 66.5% of women and 47.7% of men^26^. In another study, female sex was similarly associated with a higher prevalence of functional digestive disorders^27^. In our study, female sex was again identified as a risk factor for IBS in smokers.

A population-based study of the effects of sex and age on IBS incidence found that IBS became significantly more common with age^28^. Another study found that subjects younger than 30 years were more likely to have IBS^29^. In our study, the mean age was higher in IBS(+) smokers than in IBS(-) smokers.

A study of the role of smoking in FGIDs found that the risk of functional dyspepsia was 50% higher in current smokers than never smokers, but the associations between smoking and other FGIDs were weak^30^. The associations between smoking and overlaps of gastroesophageal reflux, functional disorders, and IBS in Japanese adults were significantly stronger in those who smoked one or more packs per day than in those who smoked less^30^. In our study, overlaps were not considered. Still, there were significant differences in the years of smoking and the FTND score between IBS(+) and IBS(-) smokers, and smoking cessation was associated with a substantial decrease in GI symptoms. A linear nicotine dependence trend was shown in the difference between patients with and without IBS, regardless of the difference between dependence groups.

Another study found that the frequency of abdominal pain, as defined in Rome IV, was the most important contributor to the reduction in the estimated prevalence of IBS from 11.7% using Rome III to 5.7% using Rome IV^31,32^. This means that half of the patients diagnosed with IBS by Rome III would not be diagnosed with IBS by Rome IV. The prevalence of IBS found in studies using Rome IV would be expected to be lower than that in studies using Rome III. Hence, the prevalence of 18.1% in our study might be considered high in the Rome IV because the global prevalence of IBS was detected with the Rome II and Rome III criteria, mostly. As there are no previous studies that report the global prevalence of IBS using the Rome IV criteria, the high prevalence of IBS reported in our study is uncertain. Hence, there is a need for general population-based studies to compare the new prevalence of IBS by the Rome IV criteria with that obtained using older versions of these criteria.

### Limitations

This study has limitations. First, the study population consisted of smokers seeking treatment for smoking cessation and may have had higher dependence scores than those in the general population, which could have introduced a degree of selection bias. Second, the diagnosis of IBS was based on the Rome IV criteria and medical records without endoscopy.

## CONCLUSIONS

The findings of this study indicate that IBS is as likely in heavy smokers as in other smokers, although IBS(+) smokers had a higher dependence score and had smoked for longer. Therefore, future studies comparing patients with IBS between smokers and never smokers would be convenient. Similar to the studies performed in the general population, female sex was an independent risk factor for IBS in smokers. This study contributes to the literature because it is the first to evaluate treatment-seeking smokers for IBS and GI symptoms based on the Rome IV criteria before and after cessation treatment.

## Data Availability

The data supporting this research are available from the authors on reasonable request.
